# Supplementary material for: Are carbon emissions trading and green financial instruments synergistic? -Comprehensive quantitative research based on content analysis
Source: PLoS One. 2024 Mar 7;19(3):e0298601. doi: 10.1371/journal.pone.0298601 (PMC10919601; doi:10.1371/journal.pone.0298601)
Supplement: S1 Data — (ZIP) [file pone.0298601.s001.zip › Data/地级市Z_F工作报告.zip!/║■─╧/╗│╗»╩╨/└┤╫╘╣1⁄2╓┌║┼í╛┬φ┐╦╩2╛▌═°í┐_2016.docx]

各位代表：

现在，我代表市人民政府向大会报告工作，请予审查，并请市政协各位委员和其他列席人员提出意见。

今年工作为“十二五”发展赢得圆满收官

2015年，我们在市委坚强领导下，紧紧围绕“一个中心、四个怀化”战略，主动适应经济发展新常态，积极应对经济下行新压力，着力化解经济工作新困难，奋力追赶全省经济发展步伐。预计全年实现生产总值1280亿元，增长8.6%，高于年初计划0.1个百分点。完成全社会固定资产投资1004.6亿元，增长18%，高于年初计划3个百分点。完成公共财政收入110亿元、地方财政收入72.81亿元，均增长10%，完成年初计划目标。规模工业增加值增长7.2%，低于年初计划4.8个百分点。实现社会消费品零售总额502亿元，增长11.3%，低于年初计划1.2个百分点。城、乡居民人均可支配收入分别达到20940元、7130元，增长9%和10%，均低于年初计划1个百分点。根据市四届人大三次会议《政府工作报告》对今年工作的部署，重点抓了“八个推进”。

(一)推进产业园区倍增计划。把产业及产业园区发展作为经济工作的第一大主战场，启动三年倍增计划，逐季逐县开展现场巡检讲评，吹响产业园区攻坚号角，全市产业园区完成基础设施建设投资42亿元，建成标准化厂房170万平方米，实现技工贸收入710亿元。出台产业引导政策，推动优势特色产业向园区集聚，建设园区技术创新平台，高新技术、电子信息、广告创意等优势特色产业发展来势向好，新增高新技术企业9家，国家高新技术企业增加到35家，省级技术中心增加到8家。强化工业经济运行调度和重点企业帮扶，选派20名处级干部进驻重点企业一对一帮扶指导，协调煤电油运气等要素供应，帮助企业解决融资、招工和拓展市场等问题。但由于经济下行压力超出预期，特别是工业品价格持续走低，加之调整结构淘汰落后产能，水电、冶金等支柱产业受到一定影响，规模工业增加值增速低于预期目标。

(二)推进中心城区提质改造。加快城区断头路拉通及改造，瑞丰路、刘塘路延长线改造基本完成，红星北路、高堰路、云集路、南环线等拓改工程即将竣工，城东、城北等高速公路主入口拓改加快推进，锦溪桥改扩建进展顺利，92条背街小巷改造完成。扎实推进城区供水管网、污水处理厂、垃圾中转站等公用设施建设，提质改造中心城区9个农贸市场。以创建国家卫生城市和省级文明城市为载体，开展以街边清洁、街头清静、街道清新为主要内容的集中整治行动，依法依规推进控违拆违工作。以入列全省新型城镇化试点市为契机，推进城乡共促、基础共享、服务均等一体化建设，市本级30个棚改项目完成投资5.67亿元，建成14个6835套，实施43个村民安置区项目，建成投用11个;加快推进县城和小城镇建设管理及综合提质，一批经济强镇、中心城镇、特色新镇加快规划和建设。

(三)推进重点项目建设。实施市级重点项目259个，正清新版GMP口服制剂生产线、华能苏宝顶风电等一批重大产业项目竣工投产，怀邵衡快铁、武靖高速、芷江机场改扩建等一批重大基础设施项目顺利推进，石煤综合利用、渝怀复线已经开工，怀芷高速即将开工。大力招商引资，全年引进产业项目195个，美达集团投资100亿元的武陵山现代商贸物流城、华美立家投资40亿元的武陵国际商贸城、浪潮集团投资6亿元的智慧怀化建设等重大项目正在建设。获批建设用地1.56万亩，征拆腾地平稳有序，重点项目建设用地保障有力。持续开展专项整治，重点工程建设环境得到优化。

(四)推进强农惠农工作。加速发展现代农业。完成粮食播面485.3万亩，总产182万吨。新建农业标准化核心示范园区120万亩，新增省级示范合作社10家，新获“三品一标”认证45个。实现农产品加工销售收入288亿元，增长19.7%。涉农电子商务平台发展到500多家，农村电子商务来势良好，溆浦县、中方县被确定为电子商务进农村示范县。着力改善农村基础条件。完成70座上型病险水库除险加固，升级改造农网6.8万户，新建和改造农村低压线路3800千米，建设高标准农田10万亩。扎实抓好精准扶贫。出台《精准扶贫攻坚行动实施纲要》，启动10项“到村到户”行动。全面开展精准复核，贫困村识别准确率100%，贫困人口识别准确率99.6%。深入开展“一进二访”活动，贫困村进村走访率、贫困户上门走访率均达到100%。全面落实主体责任，实行脱贫时间倒排、任务倒排，18.81万贫困人口稳定脱贫。

(五)推进服务业提质转型。围绕打造西南一流商贸物流基地，启动怀化铁路编组站搬迁工作，同步规划建设现代物流产业园。实施重点商贸物流项目29个，完成投资38.5亿元，为年计划的101.7%。国际汽车城建设进度加快，迎丰路与人民路、鹤洲路合围区域现代商业街建设稳步推进。淘宝电子商务平台上线运营，成为全省首家上线的地市馆，电子商务交易额达到45亿元。实施农贸市场标准化改造项目34个、万村千乡市场工程3个。围绕建设全国知名生态文化旅游目的地，出台《关于加快推进生态文化旅游业发展的意见》，实施“1354”工程，全年接待国内外游客、旅游总收入均增长15%。围绕推动各类服务业提质升级，规范和引导房地产市场平稳健康发展，妥善解决13836户办证遗留问题，销售商品房398.58万平方米，增长69.85%;成功举办房交会、汽车博览会和生态食品博览会等节会，会展营销、服务外包、健康养生等新兴服务业加速发展。由于国际原油价格下调导致石油及制品类零售额增速放缓，加之限额以上企业支撑作用有限、中小商贸企业运营成本攀升，社会消费品零售总额增速低于年初预期目标。

(六)推进改革开放和创新发展。市委确定的9大类51项改革稳步推进。市级城管31个管理机构、2291名各类人员整体下放鹤城区，长期制约城市管理的机制体制难题得到破解。下放怀化工业园区、怀化经开区市级经济社会管理权限76项，分别实行2号公章和3号公章制度。积极推动投融资体制改革，市城建投、水务投、交建投和土地收储中心四大平台加快整合。积极推行PPP融资模式，融资渠道进一步拓宽。农村金融改革取得重大进展，怀化农商行等6家农商行顺利组建，沅陵县跻身全国第二轮农村金融改革试验区。靖州向乡镇简政放权、麻阳金融产业扶贫、会同县林业投融资体制和“互联网+民生服务”改革形成可复制经验。溆浦县入列省直管县试点。市本级政府机构改革基本完成，县市区政府机构改革加快推进。乡镇区划调整基本完成，撤并乡镇93个，超省定任务5个。完成市政府部门“四张清单”梳理编制，行政审批“两集中两到位”改革全面实施。不断深化开放合作，长沙海关驻怀化监管机构筹备办已经挂牌。全年招商引资到位资金486亿元。怀化农业科技园、靖州现代农业示范区获批国家级园区。大力推动大众创业、万众创新，新增市场主体2.7万户，增长5%。

(七)推进生态怀化建设。开展市区空气质量提升行动，市区空气环境质量不断提高。加强水环境污染治理，完成重金属污染治理项目37个，启动村镇集中式饮用水源保护区划定工作，水质达标率100%。加快“美丽乡村·幸福家园”建设，创建“秀美村庄”示范村95个。完成高铁高速沿线造林2.6万亩、幼林抚育10.5万亩、封山育林25.9万亩、国省干道两旁绿化提质338公里，绿化裸露山地4.7万亩。启动农村环境综合整治全市域覆盖工作。实施重点减排项目120个，加快淘汰落后产能和黄标车，全面完成省政府下达的主要污染物减排目标。深入推进生态创建，中方县、芷江、会同县、洪江区成功创建省级生态区，国家级生态乡镇达到44个，居全省首位。创建全国医疗废物综合管理示范城市通过验收。

(八)推进各项社会事业。省市确定的17项29件为民办实事任务全面完成。民生支出占财政总支出比重提高到77.36%。新增城镇就业3.67万人，农村劳动力转移就业5.28万人，城镇登记失业率控制在4.5%以内。五大保险登记参保达548万人次。城乡低保人数达到46.3万人，发放低保资金7.2亿元，增幅排全省第一。在全国率先启动550个空巢老人和留守儿童关爱服务中心建设，改扩建乡镇敬老院9所。开工保障性住房4.3万套，改造农村危旧房2.1万户。解决44万农村人口饮水安全问题。建成义务教育合格学校90所、农村公办幼儿园26所、农村教师公租房4928套，改造农村中小学危房9.5万平方米。在全省率先推行新农合大病保险制度和医疗应急救助，农村孕产妇住院分娩基本医疗实行全免费，新农合参合率98.4%。诚信计生工作在全国会议上得到推介。成功承办湖南省纪念抗战胜利70周年大会、第五届中国·芷江和平文化节和纪念向警予同志诞辰120周年等重大活动。群众性文化活动和全民健身运动蓬勃开展。抓好耕地保护，耕地占补平衡。畅通信访渠道，加强矛盾排查调处，完善社会治安防控体系，强力开展打击涉黑涉恶势力及其保护伞、黄赌毒犯罪、多发性侵财犯罪、非法集资四大专项行动，公众安全感得到提升。扎实开展安全生产大检查、大排查、大整治，事故起数和死亡人数实现“双下降”。食品药品安全保障有力，未发生一起安全事件。深入贯彻依法治国基本方略，建立健全科学民主决策机制，办理人大代表建议184件、政协委员提案151件。加强审计监督和行政监察，政风行风持续好转。推进国防教育和国防动员工作，预备役部队和民兵建设水平不断提升。支持工会、共青团、妇女儿童、科学普及等各项事业发展，统计、档案、水文、气象、地方志、防震减灾、外事侨务、民族宗教、检验检疫、老龄老干、优抚安置、质量技术监督、住房公积金管理等工作取得新成效。

各位代表!

今年主要目标任务的完成，标志着“十二五”规划胜利收官，使我们站在更高的发展平台和起点上。

五年来，经济实力实现了新提升。地区生产总值年均增长10%，2015年达到1280亿元，是2010年的1.9倍。公共财政收入年均增长13.6%，2015年达到110亿元，是2010年的1.9倍。累计完成全社会固定资产投资3700亿元，是“十一五”时期的3.32倍，年均增长21.2%。社会消费品零售总额年均增长14.5%，2015年达到502亿元。相比五年以前，今天的怀化，经济基础更加厚实，进入了一个规模加速扩张、质量不断提升的关键时期，蕴含着经济社会发展的勃勃生机和巨大潜力。

五年来，交通条件实现了新突破。怀化进入“高铁时代”，高铁里程达178公里，域内设有4个站点，均居全省首位;杭瑞、沪昆、包茂、娄怀、绕城高速公路在境内纵横交错，通车里程680公里，居全省首位;芷江机场开通北京、上海、广州、深圳等5条航线通达9座城市;国省干线改造验收、县乡村道升级为国省道均居全省之最。相比五年以前，今天的怀化，立体交通网络更加便捷，可以全方位实现南下北上、东进西出、通江达海，在五省边区具有独特的区位交通优势。

五年来，城乡面貌实现了新改善。中心城区建成区面积拓展到64平方公里，常住人口达到60万，城镇化率提升到43%。所有乡镇和99%的行政村通水泥(沥青)路，解决165万农村人口饮水安全问题，实施村容村貌整治工程200项。城、乡电网改造率分别达到100%和97.3%，互联网普及率达到38%，在全省率先实现宽带基础网络全光网化。森林覆盖率提高到70.8%，环境质量不断改善。相比五年以前，今天的怀化，现代都市气息更加浓郁，青山绿水和蓝天白云辉映着美丽乡村、幸福家园，正在向城乡一体化迈进。

五年来，社会事业实现了新发展。2015年城、乡居民人均可支配收入分别是2010年的1.69倍、2.02倍，年均分别增长11%、15%。贫困人口从128万减少到56.42万，贫困发生率由25.1%下降到10.8%。新增城镇就业17.9万人，农村劳动力转移就业稳定在95万人左右。建设保障性住房11.3万套，实施农村危房改造7.8万户。教育文化、卫生计生、社会保障等公共服务体系不断完善。社会治安综合治理水平大幅提升，公共安全保障更加有力。相比五年以前，今天的怀化，人民群众更加安居乐业，社会文明程度进一步提升，各项社会事业全面发展，人民群众获得感、幸福感持续增强。

五年来，精神风貌实现了新转变。全面深化政治、文化、社会、生态文明等领域改革，发展活力不断增强。不断扩大对外开放，怀化背靠雪峰、融入全国、放眼世界，人民群众视野更加开阔。深入开展党的群众路线教育实践活动和“三严三实”专题教育，大力开展“宣战庸懒散、提振精气神”作风整顿，“四风”积弊得到有效整治，干部队伍作风明显改善。扎实推进文明乡村、文明社区等创建，社会风气明显净化，人民群众勤劳创业、积极创新的氛围更加浓厚。2015年全市综合民调全省排名第二，社会治安综合治理连续两年排名第一。相比五年以前，今天的怀化，社会大局更加稳定，干部群众思想观念进一步解放，干事创业心齐气顺，为创造怀化更加美好的明天，汇集了更加强劲的正能量。

各位代表!

五年来，我们共同见证了怀化的发展和变化，共同见证了怀化的成长和进步。五年的蝶变蕴含艰辛，五年的成果令人振奋。这其中，最需要我们牢记的，是长期以来关心支持怀化发展的各级领导、各界朋友，是为怀化的发展进步尽心竭力、挥洒汗水的历任前辈和同仁，是大力支持怀化改革建设的市人大代表、市政协委员和各民主党派、工商联及怀化籍在外人士，是辛勤工作在全市各条战线上的广大干部群众，以及驻怀人民解放军、武警消防官兵和公安政法干警。在此，我代表市人民政府，向大家表示衷心的感谢并致以崇高的敬意!

我们也清醒地认识到，当前政府工作与全市人民的期待还有差距，经济社会发展还面临不少困难和问题。主要是：经济总量不大，市场主体不强，经济转型发展、结构调整优化的任务还相当繁重。体制机制制约仍然突出，影响经济社会发展的一些深层次矛盾仍未消除。教育卫生、居民安置等事关民生民利的问题仍然较多，脱贫攻坚和全面小康的任务还很重。一些部门工作落实不力、服务效率不高、为政不勤不廉的现象仍然存在。对于这些问题，我们一定采取有力措施，认真加以解决。“十三五”蓝图为明年开局指明奋进方向

“十三五”时期，是实现第一个百年奋斗目标、全面建成小康社会的决胜阶段。当前，我市正处于工业化、城镇化加速推进阶段，农业现代化持续转型，信息化日新月异，绿色化潜力无限。加之我市交通、区位、生态、文化等优势领域空间广阔，城市建设、产业发展、脱贫攻坚等短板方面潜力巨大，怀化拥有更为广阔的发展空间。

当前和今后一个时期，政府工作的总体思路是：高举中国特色社会主义伟大旗帜，全面贯彻党的十八大和十八届三中、四中、五中全会精神，以马克思列宁主义、毛泽东思想、邓小平理论、“三个代表”重要思想、科学发展观为指导，深入贯彻习近平总书记系列重要讲话精神，坚持“四个全面”战略布局，贯彻创新、协调、绿色、开放、共享发展新理念，坚持以提高经济发展质量和效益为中心，坚持“三量齐升”、“五化同步”，奋力实施“一极两带”和“一个中心、四个怀化”战略，确保如期全面建成小康社会，谱写建设富饶美丽幸福新湖南的怀化篇章。

主要预期目标是：地区生产总值年均增长9%以上，城乡居民人均可支配收入比2010年翻一番，公共财政收入年均增长11%以上，全社会固定资产投资年均增长16%以上，社会消费品零售总额年均增长12%以上，规模工业增加值年均增长8%以上，进出口总额年均增长20%以上，中心城区建成区面积突破80平方公里，人口达到80万，城镇化率达到55%。森林覆盖率和蓄积量稳步提高，主体功能区布局和生态安全屏障基本形成;国家环保模范城市、国家森林城市、省级园林城市成功创建，生态文明建设走在全国前列;全面完成脱贫攻坚任务，人民群众的获得感和幸福感明显增强。

今后五年，要把握高铁经济这个重大机遇。“十三五”时期，是我市重要战略机遇期、区域发展黄金期、转型升级关键期、创新活力迸发期，特别是伴随沪昆高铁的通车和重点推进的张吉怀高铁、怀邵衡快铁建设，怀化“大字”型高铁枢纽即将成型。我们必须打破固化的空间距离、板块分割和行政区划观念，增强经济一体化、区域一盘棋意识，以全新的视角、思路谋划发展。高水平、前瞻性地做好战略规划，在“一极两带”建设中，结合自身资源禀赋、城镇特质，明确发展定位和目标，打造支撑可持续发展的城镇体系和产业体系。加强高铁沿线城镇、产业以及人流、物流的规划和引导，着力构建完整配套的政策体系。我们必须加快适应高铁经济时代的新理念，切实做到行政提效、服务提质，以更加开放创新、文明高效的理念，把怀化打造成为沪昆高铁经济带的重要支点城市，最大限度释放高铁红利。

今后五年，要把握产业发展这个艰巨任务。各地发展的经验反复告诉我们：抓好产业是打基础、利长远、增后劲的头等大事，产业不发展就谈不上竞争力。未来五年，我们必须坚持把产业及产业园区的发展放到经济工作高于一切、先于一切、重于一切的位置，以只争朝夕的紧迫感和锲而不舍的坚定决心，盯紧抓实“第一大主战场”不放松、不动摇，为稳增长提供强大的动力引擎。坚定不移地把新型工业化作为富民强市的第一推动力，建设怀黔千亿工业走廊，打造一批百亿产业、百亿园区、百亿基地。大力发展现代农业，着力改善农业基础设施，加快优势农产品基地建设，扶持特色农业、生态农业、休闲农业和都市农业发展，做大做强一批骨干龙头企业。加快发展现代服务业，突出抓好电子商务、现代金融、广告创意、信息服务等生产性服务业，加快发展商贸服务、社区服务、旅游文化、住宅产业等生活性服务业，逐步形成规模较大、结构优化、布局合理、效益提升、后劲增强的产业发展格局。

今后五年，要把握项目建设这个关键抓手。抓发展就要抓项目，没有项目，发展就是纸上谈兵。充分发挥自身资源优势，精心谋划一批关联度高、带动性强的战略性大项目、好项目。紧盯国家和省里政策投资动向，争取落户一批对经济发展有支撑引领作用的重大项目。继续强化责任考核、警示约谈、项目巡检，加快推进一批重大项目建设。我们每一位干部，都要把项目建设当作最硬的抓手、最实的任务、最大的舞台，把心思集中到大干项目上，把本领体现在干大项目上，把目标锁定在干成项目上，充分发挥项目投资对经济发展的拉动作用。

今后五年，要把握精准脱贫这个政治责任。目前怀化还有1237个贫困村、56.42万贫困人口。必须坚持把脱贫攻坚作为重大政治责任，按照中央“五个一批”要求，精准施策，精准发力，认真落实10项“到村到户”行动，着力扶持具备劳动能力的贫困人口生产和就业，积极推动贫困群众生态移民，不折不扣落实低保、五保贫困人口政策兜底，切实加大因病致贫返贫群众的医疗救助力度。顺应全市人民共享发展成果、过上美好生活的期盼，把更多的公共资源向民生倾斜，大力发展各项社会事业，着力办好一批民生实事，不断提高社会保障水平，真正让老百姓的生活年年都有新变化，一年更比一年好。

今后五年，要把握改革创新这个根本动力。破解当前面临的各种难题，必须突出供给侧结构性改革，抓住牵一发动全身的关键环节，推出一批具有重大牵引作用的改革创新举措。下大力气推进简政放权、现代产权制度、投融资体制、财税体制、人才培养引进使用机制等重点领域和关键环节的改革，加快形成适应新常态的体制机制和充满活力、富有效率的发展环境。强化企业创新主体地位，加强核心关键技术攻关，深化产学研合作，加快建设一批研发中心、孵化基地、实验室，积极争取科技重点项目和重大专项支持，着力培育一支优秀的创新型人才队伍，全面优化创新创业环境，努力在全社会形成创造能量充分释放、创新成果不断涌现、创业活动蓬勃开展的生动局面。

今后五年，要把握干部作风这个坚实保障。作风关系行政效能，关系政府形象，关系人心向背。紧紧围绕未来五年目标，切实增强工作针对性和实效性，增强政策措施可行性和操作性。实行严格的考核制度，做到责任明确、措施具体、考核科学、奖优罚劣，确保每项工作有部署、有检查、有落实、有评价。在全市上下树立敢负责、会干事、能吃苦的工作作风，既要为具有这种作风的干部撑腰壮胆，更要让这样的干部脱颖而出，在干事创业中形成敢抓落实、狠抓落实、会抓落实的优良作风。

2016年是全面建成小康社会决胜阶段的开局之年，也是实施“十三五”规划的第一年，必须牢牢把握稳增长、抓重点、惠民生、防风险的总体要求，全力以赴开好局、起好步。主要目标任务是：地区生产总值增长9%以上，公共财政收入和地方财政收入均增长11%以上，规模工业增加值增长8.5%以上，全社会固定资产投资增长17%以上，社会消费品零售总额增长12%以上，城、乡居民人均可支配收入分别增长9%、10%以上，单位GDP综合能耗及主要污染物减排完成省里考核目标。为此，要着力抓好七个方面工作。

(一)围绕实现“三年倍增”，突出抓好产业及产业园区发展。强化工业经济龙头带动作用。完成园区基础建设投入50亿元，新建标准化厂房135万平方米。创新园区管理体制、运行机制和人才引进、薪酬激励制度，增强园区发展活力。积极探索飞地经济模式，加快建设以怀化工业园区为龙头的怀黔千亿工业走廊，努力形成工业园区竞相发展的良好势头。全年产业园区技工贸总收入、财税收入、固定资产投资、招商引资增长25%。积极对接“中国制造2025”、“互联网+”行动计划、制造强省“1274”行动，突出电子信息、现代制造、生物医药、新材料、新能源、节能环保等重点产业，培育壮大骨干龙头企业，推进同产业链企业分工合作，促进优势特色产业集聚发展。落实各项惠企政策，继续实行干部进驻企业定点帮扶制度，帮助企业降低成本，加强生产要素协调供应，促进企业正常开工、高效运行、增产增效。加快推进农业现代化。抓好优势农产品基地、全国农产品加工创业基地、国家农业科技示范园基地建设，完善农业专业化服务体系，促进农业与二三产业融合发展。稳步扩大粮油、果蔬茶、畜牧水产等优势农产品基地规模，新增市级以上农业产业化龙头企业15家，力争农产品加工销售收入增长19%。推进碣滩茶等特色农产品品牌整合，打造一批怀化农业品牌，争创农产品中国驰名商标1—2个。加快培育专业大户、家庭农场、农民专业合作社等新型农业经营开发主体。实施“百片千园万名”科技兴农工程，加快种养殖业新品种新技术推广步伐。推进农机办点扩面，力争农机总动力增长6%。健全农产品质量安全体系，加强重大动植物疫病防控。提升服务业发展质量和效益。推进一批商贸物流重大项目建设，促进本土重点企业做大做强，培育一批具有核心竞争力的大中型商贸物流企业，促进传统商贸物流业转型升级。大力发展电子商务，努力创造农村电商平台怀化特色，力争走在全省前列。大力实施生态文化旅游“1354”工程，深入挖掘民俗文化、稻作文化、和平文化、红色文化、高庙文化等特色文化，打造精品线路，加快融入张吉怀精品生态文化旅游经济带，力争接待国内外游客3800万人次，旅游总收入230亿元。办好车博会、农博会，积极筹办武陵山片区健康产业博览会、家具博览会等新型展会，促进会展业规范有序发展。引导房地产市场健康稳步发展，打通供需通道，加快消化库存。

(二)围绕加快新型城镇化，突出抓好城乡基础设施建设。认真贯彻落实中央、省委城市工作会议精神，全力加快新型城镇化步伐。改革完善城镇规划，完善城镇总体规划、专项规划和控制性详细规划，促进经济社会发展规划、城乡规划、土地利用规划、生态环境保护规划等多规合一。围绕打造鹤中洪芷生态城镇群，坚持产城融合发展，优化中心城市布局，构建“一核三轴六组团”的空间格局。突出建设高铁新城，深化城市综合提质，推进城区道路、公用设施、主题公园、环城生态圈等工程建设，加快形成“一圈两水四山七园”的生态布局，打造中心城市新亮点。推进智慧城市建设工程，探索数字化城市管理模式，促进城市管理工作全面升级。加大拆违控违力度，坚决遏制乱搭乱建。继续抓好清街集中整治行动，突出整治脏、乱、闹问题，实现街边清洁、街头清静、街道清新。推动国家卫生城市、省级园林城市创建工作，确保省级文明城市创建成功。深入实施省“百城千镇万村”工程，全面开展农村环境综合治理，美化、绿化、净化农村人居环境，大力建设“美丽乡村·幸福家园”。完善城乡交通网络，加快怀化编组站搬迁和怀邵衡快铁、武靖高速、怀化综合客运枢纽站、城市公交站场建设，促成张吉怀高铁及靖州至黎平高速公路尽早开工，推进G209会同绕城公路、S317沅陵苦藤铺至白田公路等国省干线公路建设，完成S332进马江至新建公路二期工程、G319沅陵过镇改线工程等国省干线项目。

(三)围绕脱贫攻坚，突出抓好到村到户行动。把脱贫攻坚作为重大政治任务和重大发展机遇，确保18.81万贫困人口稳定脱贫。着力改善贫困地区基础条件。大力实施农村公路建设、安全饮水、环境治理等工程，完成所有贫困村村级道路建设，实施500公里农村公路窄路加宽工程和500公里安全防护工程。启动363个病险水库除险加固和6个中小河流治理项目，完成农村安全饮水工程423处，解决33.8万人饮水安全问题。切实保障贫困地区群众生活。深入推进新居、安居、宜居工程，启动30个整村推进特色村落项目，着力改善和解决3万贫困人口住房问题。加快市级生态移民新村建设，实施13个县级易地扶贫搬迁工程。推进162个行政村卫生室建设，对因病致贫农户实施医疗救助。更加注重对特定人群特殊困难的精准帮扶，确保3.78万贫困人口通过政策兜底脱贫。不断增强贫困地区自我发展能力。依托贫困地区资源禀赋和产业基础，抓好贫困村特色产业培育、产业示范基地建设和生态文化旅游开发。以建设全省教育精准扶贫试点市为契机，着力改善农村办学条件，落实贫困学生就学补助，抓好技术技能培训，提高贫困人口创业就业能力。不断完善农村金融服务体系，创新金融产品，强化农村商业银行服务功能，扩大信贷支持覆盖面。

(四)围绕建设绿色怀化，突出抓好生态保护和环境治理。强化绿色发展理念，划定耕地、林地和森林三条生态保护红线，更加自觉地保护生态、节约资源、改善环境。大力实施“青山工程”，加强森林公园、自然保护区、风景名胜区、生态公益林的建设和保护，积极争取国家天然林保护工程，新增国家级森林公园2个。继续推进高铁高速公路沿线绿化提质。加快淘汰一批小型竹木加工企业。大力实施“碧水工程”，抓好五强溪水库、溆浦思蒙等重要水域和湿地保护，新增国家级湿地公园1个。更大力度推进太平溪、氵舞水河综合治理，切实抓好中心城市和县城饮用水源地保护及备用水源建设，加强村镇集中式饮用水源保护区划定和保护工作。大力实施“蓝天工程”，持续推进扬尘污染、燃煤锅炉(窑炉)、烟花爆竹燃放等集中整治行动，有效控制城市雾霾、噪声污染，全年空气质量优良率达80%以上。大力实施“绿地工程”，加强固体废物污染防治，推进污染土壤修复治理，建设一批城镇公园、小游园和休闲广场。加强资源能源节约集约利用，实行能源和水资源、建设用地等总量和强度双控行动，降低资源能源消耗强度，推动形成绿色生产方式和消费方式。

(五)围绕破除体制机制障碍，突出抓好重点领域改革创新。继续深化行政审批制度改革，落实“四张清单”制度，取消和下放一批市级审批事项。加快园区体制改革，建立独立的市场化投融资平台，推动实施飞地经济、园政合一试点。积极抓好国企国资改革，实现经营性国有资产监管全覆盖。健全政府投资项目管、评、审分离的监管机制，理顺市区财政体制。继续抓好沅陵县发展新型农村合作金融组织试点和麻阳县全国农村信用体系建设试点。协调推进市县公务用车改革。创新农村产权抵押贷款和融资担保模式改革。建立和完善市级以上科技创新平台3个，高新技术企业增加到40家，组织实施一批科技创新和成果转化专项。大力推进“大众创业、万众创新”，加快出台科技服务业发展政策措施，重点支持文化创意孵化园、大学生创业园建设。落实湘黔两省高铁经济带的合作框架协议，支持大(龙)新(晃)经济协作示范园发展。积极促进长沙海关驻怀化监管机构办理海关业务，加快启动铁路口岸、公路口岸和公共保税物流仓规划设计。突出领导招商、产业链招商、小分队招商、以商招商，主动对接央企和国内500强企业，引进一批有重大影响力和核心竞争力的战略投资者，全年招商引资到位资金420亿元。

(六)围绕保障和改善民生，突出抓好民生实事。加大财政投入，加强检查调度，集中力量办好省市确定的重点民生实事，着力解决就业、教育、医疗等一批热点难点问题。推动实现更高质量的就业，加快公共就业服务平台标准化建设，促进高校毕业生、农村转移劳动力、城镇困难人员和退役军人等群体就业。全面推行农民工保障金制度，健全劳动监察和争议处理机制，构建和谐劳动关系。新增农村劳动力转移就业4.7万人、城镇就业3.5万人，城镇零就业家庭实现动态清零。强化社保扩面征缴主体责任，做好失地少地农民社会保障和社会保险基金监管工作。提高城乡低保、农村五保供养水平，抓好乡镇敬老院建设和管理，继续推进空巢老人和留守儿童关爱服务中心建设。积极探索医养结合模式，不断提高养老服务水平。支持发展社会福利和慈善事业。建设保障性住房6.1万套，实现廉租房与公租房并轨。强化住房公积金的住房保障作用。

(七)围绕促进公共服务均等化，突出抓好各项社会事业。坚持教育优先均衡发展，深入推进城区教育发展五年行动计划，新增城区义务教育学位7450个。全面完成农村中小学危房改造、义务教育合格学校和教师公租房建设任务。新建农村公办幼儿园25所、普惠性民办幼儿园51所。推动芷江师范一期工程建成使用，支持怀化工业中专申创湖南卓越职业学校。深化公立医院改革，建立疾病应急救助制度，加强药物供应保障体系建设，实现基本药物制度城乡全覆盖。按照“互联网+医疗”模式，逐步建立区域医疗信息系统，推动健康大数据应用。发展中医药事业。坚持计划生育基本国策，全面落实一对夫妇可生育两个孩子政策，提高生殖健康、妇幼保健、托幼等公共服务水平。推进公共文化服务体系建设，实施农村广播村村响等文化惠民工程。广泛开展群众文化活动，鼓励和扶持文艺精品创作。加强文化市场监管和广电新闻出版管理。重视文化遗产保护工作，支持侗寨申遗和侗族苗族文化生态保护试验区申报工作。依托通道转兵旧址，积极承办红军长征80周年纪念活动。支持民族地区用好民族政策，办好新晃60周年县庆。发展体育产业，促进体育消费，开展全民健身运动。做好第三次农业普查工作。加强双拥共建、国防动员、民兵预备役等工作，推动军民深度融合发展。落实社会稳定风险评估制度，坚持用法治思维破解维稳难题，有效防范、化解、管控各类风险。压实信访工作责任，加强乡村两级人民调解组织建设，有效化解矛盾纠纷。深入推进社会治安防控体系建设，广泛开展平安创建活动，严密防范和依法惩治各类违法犯罪，有力维护社会稳定。重视网络舆情监督管理，提高新媒体时代舆情引导水平。守住公共安全底线，采取更坚决措施，全方位强化安全生产，全过程保障食品药品安全。完善各类公共事件应急预案，加强应急救援力量，提高应急能力。统筹抓好民族宗教、老龄、台湾事务工作，加大监察、审计工作力度，发展妇女儿童事业，促进地方志、档案、保密、气象、水文、防震减灾、检验检疫等各项事业全面协调发展。坚持更高标准和要求加强政府自身建设

实现“十三五”发展目标，做好明年各项工作，必须以更高标准和要求持续加强自身建设，努力争取政府工作新作为。

努力建设依法行政重诚信的政府。不论哪一级人民政府都必须按规矩办事。如果不讲规矩，政府就会丧失公信力，工作上就会引发很多矛盾和问题，陷入被动局面。要严格按照法定权限、法定程序行使职权、履行职责，坚持运用法治思维、法治方式推动工作、化解矛盾。必须严格执行政府权力清单制度，做到法定职责必须为、法无授权不可为，促进严格执法、文明执法、公正执法相统一。要坚决贯彻落实市委决策部署，严格执行人大及其常委会的决议、决定，认真办理人大代表议案、建议和政协委员提案，自觉接受人大的法律监督、工作监督和政协的民主监督。要强化政府诚信意识，坚决兑现政府各项承诺。凡是向人民群众承诺了的事情，都要一抓到底、坚决兑现;凡是向企业和投资者明确了的政策和服务，都要一一落实、条条算数，真正做到言必行、行必果。

努力建设忠诚为民办实事的政府。怀化作为欠发达地区，老百姓生活水平还不高，政府工作必须把老百姓对美好生活的向往作为奋斗目标，把老百姓关心的问题作为发力的重点。在政府重大决策上要坚持民意导向。谋发展、作决策始终以老百姓的意愿为先、利益为重，坚持问政于民、问需于民、问计于民，落实重大决策调查研究、专家咨询、风险评估、公示听证、合法性审查等制度，使政府决策贴近民情、顺应民意、合乎民心。在政府工作部署上要强化民本意识。始终牢记老百姓的利益无小事，把工作着力点真正放到服务基层、服务企业、服务群众上，带着深厚感情和强烈责任为老百姓办实事、解难题。在公共资源配置上要深植民生情怀。财政资金使用、政府项目安排重点向基层一线倾斜，把公共资源用到老百姓最期待、最需要的地方，让发展成果真正惠及普通老百姓，使政府作为切实体现到改善民生上来。努力建设高效服务讲责任的政府。在过来政府的工作中，一定程度存在服务意识不强、行政效率不高的问题。与发达地区相比，工作节奏总是慢半拍，甚至存在不推不动、推而不动的现象。要坚持简政放权，加快理顺政府与市场、社会的关系，最大限度精简审批项目，精简审批程序，精简审批时间，不断激发市场和社会的活力。要坚持完善制度，加快完善目标责任体系，健全工作考核方式，创新工作推进责任机制，确保市委作出的决策部署和市政府要抓的各项工作，定一件办一件，办一件成一件。要坚持改进作风，大力弘扬求真务实作风，讲实话、重实干、办实事、求实效，力戒形式主义和官僚主义，集中更多的时间和精力解决实际问题，推动工作落实。要坚持行政问责，推行岗位责任制、首问负责制、服务承诺制、限时办结制等制度，切实增强各级政府服务发展的责任，真正以促进发展为首要职责，做到推动工作决不放松、促进发展决不懈怠、服务群众决不马虎。

努力建设攻坚破难敢担当的政府。当前，我市正处于爬坡过坎的重要关口，尤其需要方方面面心连心、肩并肩，携手共渡难关，在挑战、压力、困难面前敢于担当。面对产业发展、城市建设、项目推进等重点工作，要拿出魄力、奋力作为。面对抗灾抢险、专项整治、征拆安置等急难问题，要主动上前、攻克难关。面对全面小康、深化改革、脱贫攻坚等重大任务，要矢志不移、负重前行。各级政府必须拿出坚定的决心、有力的举措、科学的办法，雷厉风行地解决遇到的各种困难和矛盾。

努力建设严以律己守清廉的政府。加快怀化经济社会发展，必须有一个风清气正的良好环境。必须自觉践行“三严三实”，坚持以严的标准和实的要求，努力做到清正廉洁。要切实管好权力。严格在法律约束下用权，在制度笼子里用权，做到政府职权法授、程序法定、行为法限、责任法究。要切实管好钱财。健全严格的财务预算、核准和审计制度，加强财政资金、公共资产管理;加强政府投资项目的财政监管，防范政府债务风险;坚持勤俭办一切事情，力倡艰苦奋斗，力戒奢侈浪费，进一步降低行政运行成本。要切实管好队伍。认真履行“一岗双责”，强化“两个责任”，决不能以经济工作取代政治工作，以业务工作取代党务工作;深入开展党纪政纪法纪教育和警示教育，加强廉政文化建设，增强抵御诱惑、守住底线、廉洁自律的定力;强化行政监察，严格行政追责，坚持政府工作人员贪腐行为“零容忍”，做到发现一起，查处一起，决不姑息。

各位代表!“十三五”的蓝图已经绘就，新一年的工作即将铺开。只要我们在中共怀化市委的坚强领导下，坚定信心，同舟共济，斗志昂扬，奋力拼搏，就一定能够汇集推动怀化发展的强大力量，就一定能够完成明年经济社会发展各项任务和“十三五”宏伟目标，就一定能够夺取全面建成小康社会的新胜利!
